# Serological Immunoassay for Hansen’s Disease Diagnosis and Monitoring Treatment: Anti-Mce1A Antibody Response Among Hansen’s Disease Patients and Their Household Contacts in Northeastern Brazil

**DOI:** 10.3389/fmed.2022.855787

**Published:** 2022-06-09

**Authors:** Filipe Rocha Lima, Fred Bernardes Filho, Vanderson Mayron Granemann Antunes, Jaci Maria Santana, Regina Coeli Palma de Almeida, Diana Mota Toro, Vinicius Fozatti Bragagnollo, Gabriel Martins da Costa Manso, Natália Aparecida de Paula, Eliracema Silva Alves, Lee W. Riley, Sérgio Arruda, Marco Andrey Cipriani Frade

**Affiliations:** ^1^Healing and Hansen’s Disease Laboratory, Ribeirão Preto Medical School, University of São Paulo, São Paulo, Brazil; ^2^Dermatology Division, Department of Internal Medicine, National Referral Center for Sanitary Dermatology and HD, Clinical Hospital of the Ribeirão Preto Medical School, University of São Paulo, São Paulo, Brazil; ^3^Department of Clinical, Toxicological, and Bromatological Analyses, School of Pharmaceutical Sciences of Ribeirão Preto, University of São Paulo, São Paulo, Brazil; ^4^Directorate of Unit and Health Care Surveillance, HD Control Program, State Department of Health, Piauí, Brazil; ^5^Federal University of Piauí, Piauí, Brazil; ^6^Division of Infectious Diseases and Vaccinology, School of Public Health, University of California, Berkeley, Berkeley, CA, United States; ^7^Advanced Public Health Laboratory, Gonçalo Moniz Institute, Oswaldo Cruz Foundation, Salvador, Brazil

**Keywords:** serological, biomarkers, Hansen’s disease, Mce1A protein, antibodies

## Abstract

Hansen’s disease (HD) is an ancient disease, but more than 200,000 new cases were reported worldwide in 2019. Currently, there are not many satisfactory immunoassay methods for its diagnosis. We evaluated antibodies against Mce1A as a promising new serological biomarker. We collected plasma from new cases, contacts, and endemic controls in the city of Parnaíba and treated patients at Carpina, a former HD colony in Piauí state, northeastern Brazil. Receiver operating characteristic (ROC) curves were used to assess the assay thresholds, specificity and sensitivity of the IgA, IgM, and IgG antibodies against α-Mce1A by indirect ELISA and compared it with IgM anti-PGL-I and molecular diagnosis by quantitative polymerase chain reaction (qPCR). Venn diagrams were generated to represent the overlap in the antibody positivity pattern. Multivariate analysis was performed to assess the potential predictor of antibodies for the outcome of having an HD diagnosis. IgA and IgG were positive in 92.3 and 84% of patients, respectively. IgM was negative for all treated patients. IgG had a sensitivity and specificity of 94.7 and 100%, respectively. IgM-positive individuals had a 3.6 chance of being diagnosed with HD [OR = 3.6 (95% CI = 1.1–11.6); *p* = 0.028], while IgA-positive individuals had a 2.3 chance [OR = 2.3 (95% CI = 1.2–4.3); *p* = 0.005] compared to endemic controls. We found that the Mce1A antibody profile can be an excellent diagnostic method of HD. IgA is an ideal biomarker for confirming contact with the bacillus. IgM has potential in the detection of active disease. IgG antibodies confirm the performance of these serological markers in diagnosis and therapeutic follow-up.

## Introduction

HD is a disabling chronic infectious disease caused by *Mycobacterium leprae* that affects the skin and peripheral nerves (1). The disease has high morbidity, mainly due to neural involvement, which can cause permanent physical disabilities and deformities, reinforcing its social stigma (2). In Brazil, during the period without effective therapeutic treatment, HD patients were compulsorily segregated from society into HD colonies (3). The Hospital Colônia do Carpina in Parnaíba-Piauí (PI) was founded in 1931, housing approximately 300 HD patients (4, 5).

The strategy of an early diagnosis and effective treatment with multidrug therapy (MDT) is crucial for HD cure, preventing sequelae and reducing the disease stigma (6). However, the diagnosis of HD is difficult and requires qualified professionals to differentiate it from other dermatological or neurological diseases (7). The current limitations of diagnostic tests, including test accuracy, and the lack of availability of low-cost commercial kits and easy implementation in primary care health units, indicate a need for more effective tests for diagnosis monitoring treatment and assessing household transmission. In addition, there is currently no method that can diagnose all HD clinical forms (8). Thus, the absence of tests that allow for the identification of subclinical infections and mild HD contributes to the progression and spread of the disease and the inability to reach the elimination goals proposed by the World Health Organization (WHO).

There is no laboratory test capable of detecting all clinical forms of HD. The knowledge and skills required for an HD diagnosis, treatment and management by general health workers are unsatisfactory, leading to delayed diagnosis, physical disabilities, socioeconomic impairment, and continued *M. leprae* transmission (9). Bacilloscopy from slit skin smears is the standard laboratory test to detect *M. leprae*, although highly specific, has a low sensitivity and it is performed only in presumed HD cases, and is negative in the majority of initial or neural forms. Serological tests for antibody detection in HD have many limitations in diagnose of all HD clinical forms and discriminating contacts compared to patients (10). The use of cell wall antigens of the bacillus, as serological biomarker has been well-established to detect specific antibodies, such as against PGL-I or protein glycoconjugates. Although anti-PGL-I antibodies serologic evidence has very low sensitivity and low predictive value, its high correlation with high bacillary index and almost completely multibacillary clinical forms, it can be useful in HD exclusion (10, 11). The detection of *M. leprae* DNA in earlobe slit skin smears and other sites using standard PCR or quantitative PCR has also been very useful to detect asymptomatic carriers or complex cases. New screening techniques, including PCR, peripheral nerve ultrasonography and electroneuromyographic are being employed, with a diagnostic serological test in development (12).

However, there is a need for simple, low-cost diagnostic strategies to monitor treatment and assess household transmissions at primary care settings. The mammalian cell-entry 1A (Mce1A) protein, first described in *M. tuberculosis*, is present in the cell wall of *M. leprae* and it is associated with the entry of the bacillus into nasal epithelial cells and skin cells (13, 14). Previous studies have shown the potential of using serum biomarkers such as antibodies against Mce1A in the diagnosis of HD (15). Therefore, because it plays a role in the invasion and maintenance of *M. leprae* infection, Mce1A represents a potential target for the development of new diagnostic tests to diagnose HD, monitor treatment, and screen for contacts of index cases of HD. Thus, our study aimed to evaluate and compare the presence of antibodies against PGL-I and Mce1A among patients newly diagnosed with HD and their contacts in the city of Parnaíba with patients treated for HD and HD residents and inmates of Carpina Hospital. We also explored the utility of IgA, IgM, and IgG anti-Mce1A antibodies and their correlations in HD.

## Materials and Methods

### Setting and Design

A cross-sectional study was conducted at the National Reference Center in Sanitary Dermatology and HD, Clinical Hospital of Ribeirão Preto Medical School (HCFMRP-USP), University of São Paulo, which provides training in HD management for several states of Brazil (MH-Brazil Project). Volunteer subjects were recruited by convenience sampling in March 2016 during a campaign to evaluate contacts of patients in the city Parnaíba, Brazilian municipality in the state of PI, the second most populous city in the state. Treated HD patients living in a former HD colony (Colony of Carpina), PI, Brazil, were also included in this study.

### Study Population

After signing an informed consent form, the volunteers were classified into four groups: (1) new cases of HD diagnosed during active search actions in the Parnaíba Municipality (PAR-NC), (2) treated HD patients who were residents of the Carpina Colony Hospital (CAR-TP), (3) household contacts (HHC) evaluated in Parnaíba, and (4) healthy endemic controls (EC) (Table 1).

**TABLE 1:** Study population characteristics (*N* = 82).

	EC *n* = 20	HHC *n* = 17	PAR-NC *n* = 26	CAR-TP *n* = 19	*p*-value
Age, years, mean (SD)	29.5 (12.3)	42.8 (16.7)	43.9 (16.9)	58.6 (13.0)	<0.0001^a^
**Sex, *n* (%)**					
Male	7 (35)	9 (52.9)	17 (65.4)	15 (78.9)	0.03^b^
Female	13 (65)	8 (47.1)	9 (34.6)	4 (21.1)	
**Therapeutic scheme, *n* (%)**					
PB	−	−	2 (7.7)	1 (5.3)	0.009^b,c^
MB	−	−	24 (92.3)	10 (52.6)	
DDS	−	−	0 (0)	5 (26.3)	
**Clinical form, *n* (%)**					
I	−	−	1 (3.8)	−	
TT	−	−	1 (3.8)	−	
BT	−	−	1 (3.8)	−	
BB	−	−	13 (50)	−	
BL	−	−	3 (11.6)	−	
LL	−	−	7 (27)	−	
**PCR-RLEP test, *n* (%)**					
Positive	−	−	15 (57.7)	11 (57.9)	0.09^b,d^
Negative	−	−	3 (11.5)	8 (42.1)	
Ct, mean (SD)	−	−	28⋅07 (1.065)	30⋅96 (0.330)	0.03^e^

*^a^Comparison of four groups using the Kruskal–Wallis test.*

*^b^Comparison of the four and two groups using the chi-square test.*

*^c^Data not available for three volunteers in the CAR-TP group.*

*^d^Data not available for eight volunteers in the PAR-HD group.*

*^e^Comparison of qPCR-RLEP positivity between PAR-NC and CAR-TP cells using the t-test.*

*EC, endemic controls; HHC, household contacts of HD patients; PAR-NC, new cases of HD in Parnaíba; CAR-TP, patients treated in Colony Carpina; SD, standard deviation; PB, paucibacillary; MB, multibacillary; I, indeterminate; TT, tuberculoid; BT, borderline-tuberculoid; BB, borderline-borderline; BL, borderline-lepromatous; LL, lepromatous; PCR-RLEP, quantitative polymerase chain reaction-specific repetitive element; Ct, cycle threshold; IQR, interquartile range.*

### Hansen’s Disease Cases

Newly diagnosed HD cases seen at Parnaíba and Carpina were invited to participate in this study. Patients were considered eligible for inclusion in the study if their diagnosis was confirmed by clinical evaluation and serological and/or molecular exams. All cases were classified considering the guidelines adapted by Indian Association of Leprologists (16), Ridley and Jopling (17), and Congress of Madrid classifications (18), as follows: indeterminate (I), polar tuberculoid (TT), borderline tuberculoid (BT), borderline borderline (BB), borderline lepromatous (BL), polar lepromatous (LL); and according to WHO operational criteria [PB (TT) and MB (BT, BB, BL and LL)]. All newly diagnosed patients were referred to a health unit for standard MDT.

### Household Contacts

HHC was defined as volunteers residing in the same household with an index case for at least 6 months prior to diagnosis. All HHC and EC were clinically screened for signs or symptoms suggestive of HD and subjected to laboratory analysis with serological examination. Clinical examinations were performed by trained physicians and health professionals at HCFMRP-USP.

### Endemic Controls

ECs, representing community contacts, were defined as healthy individuals residing in the city Parnaiba, PI, Brazil an endemic area who had no history of diagnosis or contact with an HD. All participants reported being test-negative for the human immunodeficiency virus and did not diseases or use immunosuppressive drugs.

### Serology to Detect IgM Anti-Previous Serologic Test by Enzyme-Linked Immunosorbent Assay

Indirect enzyme-linked immunosorbent assay (ELISA) was used to measure the anti-PGL-I IgM titer of every serum sample according to a previously reported protocol (8).

### Detection of *Mycobacterium leprae* DNA by Quantitative Polymerase Chain Reaction

Total DNA extraction of earlobes and at least one elbow and/or lesion SSS sample was performed with the QIAamp DNA Mini Kit (Qiagen, Germantown, MD, cat: 51306) was performed according to the manufacturer’s protocol. DNA was used to perform PCR-RLEP according to a previously reported protocol (19).

### Serology to Detect IgA, IgM, and Total IgG Anti-Mce1A by Enzyme-Linked Immunosorbent Assay

Quantitative assessment of IgA, IgM and IgG antibodies against the Mce1A protein was performed by indirect ELISA (15). Purified recombinant Mce1A protein was provided by Dr. LW Riley (University of California, Berkeley, CA, United States). Mce1A protein (10 μg/mL) was diluted to 1:1,000 in ethanol, and 50 μL of this solution was dried overnight on polystyrene ELISA well plates (Corning^®^ Costar^®^, Sigma Aldrich, San Luis, Missouri, United States). The ELISA plates were then blocked with 100 μL of 1% BSA (Thermo Fisher Scientific, Waltham, Massachusetts, United States) and washed with PBS (Laborclin, São José do Rio Preto, SP, Brazil) containing 0.05% Tween 20 (Sigma–Aldrich, St. Louis, MO, United States) (BSA/PBS/T). Frozen serum samples were thawed and diluted 1:100 in BSA/PBS/T. Next, 100 μL of each diluted sample was added to the plates and incubated for 1 h at room temperature (RT) (18–25°C), followed by three washes with PBS/T. Next, 100 μL of 1:10,000, 1:10,000, or 1:25,000 goat-derived anti-human IgA, IgM or IgG labeled with horseradish peroxidase (Sigma–Aldrich, St. Louis, MO, United States) diluted in BSA/PBS/T was added, followed by incubation at RT for 1 h. This was followed by repeated washing with PBS/T. Then, 100 μL TMB solution (Invitrogen Life Technologies, Carlsbad, CA, United States) was added, and the plates were reincubated for 30 min at RT. Finally, the reaction was stopped with 100 μL of 2 N sulfuric acid. Reactions were read at 450 nm in a SpectraMax M3 spectrophotometer (Molecular Devices, San Jose, CA, United States). The results were recorded as the average optical density (O.D.) of triplicate samples, and the assay was repeated if the coefficient of variance was >10%. The sample index was calculated by dividing their O.D. per the established cut-off for each immunoglobulin. Indices above 1.0 were considered positive.

### Statistical Analysis

All data were analyzed by GraphPad Prism v. 9.0 software (GraphPad Inc., La Jolla, CA, United States). Statistical variations were analyzed by the Mann–Whitney and Kruskal–Wallis tests, followed by Dunn’s test. Spearman’s correlation was used to compare the immunoglobulin levels of IgA, IgM, IgG anti-Mce1A and IgM anti-PGL-I. The ability of immunoglobulin levels to discriminate HD patients from controls (EC) was evaluated by receiver operating characteristic (ROC) curves. The chi-square test was used to assess associations among categorical variables and the presence of antibodies. Comparisons of the qPCR-RLEP positive test results were performed by the *t*-test. The level of statistical significance was set at *p* < 0.05. The Venn diagrams were generated using the online tool Draw Venn Diagram^1^ to represent the overlap in the number of antibodies differentially determined by indirect ELISA in each of the comparison groups. Binomial logistic regression analysis was performed to assess the potential predictor of antibodies for the outcome of having an HD diagnosis with the jamovi project (2021). *jamovi* (Version 1.6) (Computer Software). Retrieved from https://www.jamovi.org.

### Role of the Funding Source

The funder of the study had no role in the study design, data collection, data analysis, data interpretation, or writing of the report. All authors had full access to all of the data in the study and had final responsibility for the decision to submit for publication.

## Results

### Study Population Characteristics

The study included 82 volunteers, grouped as new HD patients from Parnaíba (PAR-NC; *n* = 26; 31.7%), treated HD patients (CAR-TP) from the colony (*n* = 19; 23.2%), HHC (*n* = 17; 20.7%), and EC (*n* = 20; 24.4%). Of the 26 PAR-NC, 24 (92.3%) were multibacillary (MB). Thirteen (50%) patients were classified to have borderline borderline (BB) clinical forms of HD, 7 (27%) as lepromatous leprosy (LL), and 3 (11.6%) as borderline lepromatous (BL). Among the 19 CAR-TP patients, 10 (52.6%) received the MB scheme, and 5 (26.3%) received monotherapy with dapsone (DDS). A significant difference was observed among the ages of the volunteers from all four groups (*p* < 0.0001) and sex (*p* = 0.03) due to the inclusion of a special population with only elderly people from the colony (mean:58.6, *SD* = 13). Descriptive characteristics of the study population are summarized in Table 1. The frequency of positive PCR-RLEP tests in PAR-NC and CAR-TP was 57.7 and 57.9%, respectively, but the cycle threshold showed significant differences between these two groups (*p* = 0.03), demonstrating a decreased bacillary load after CAR-TP and/or the use of monotherapy (DDS) in the initial treatment.

### Antibodies Against Mce1a Protein Are Biomarkers for the Diagnosis and Monitoring Treatment of Hansen’s Disease Patients and Their Contacts

**FIGURE 1:**
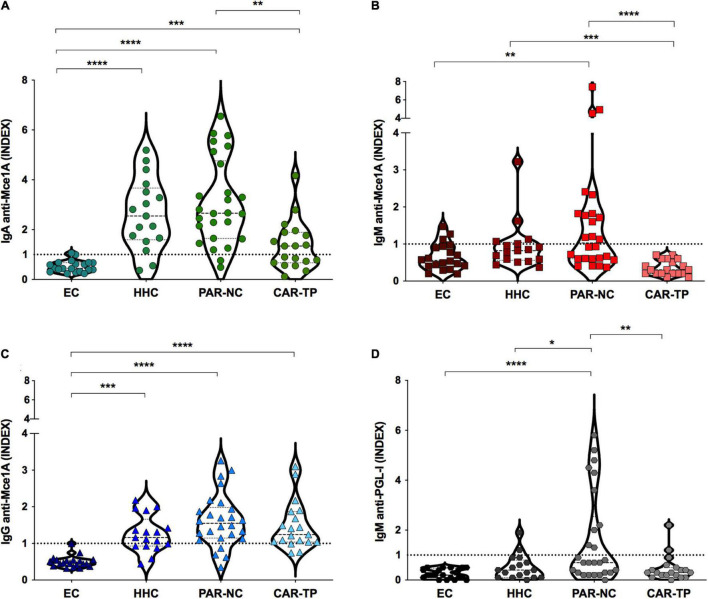
Antibodies anti-Mce1a are biomarkers for the diagnosis and monitoring of HD. IgA **(A)**, IgM **(B),** and IgG **(C)** antibody indices against Mce1A protein and PGL-I **(D)** in different groups of plasma samples tested. Statistical significance was determined by the Kruskal–Wallis test followed by the Dunn test. Data are presented as the median and interquartile range (IQR); significance was considered at *p* < 0.05, *p* < 0.01, *p* < 0.001, or *p* < 0.0001 as represented by *, **, ***, and ****, respectively. EC endemic controls (*n* = 20); HHC household contacts of HD patients in Parnaíba (*n* = 17); PAR-NC new cases of HD in Parnaíba (*n* = 26); CAR-TP patients treated in Colony Carpina (*n* = 19). The respective index was calculated by dividing the optical density (O. D 450 nm) of each sample by the cut-off, and indices above 1.0 were considered positive represented by horizontal dotted line.

The antibody profiles against Mce1A protein and PGL-I levels in PAR-NC, CAR-TP, HHC, and EC are shown in Figure 1. IgA anti-Mce1A levels were significantly higher in the HHC [median: 2.5 (IQR:1.6–3.7), *p* < 0.0001], PAR-NC [median: 2.7 (IQR:1.6–4.8), *p* < 0.0001] and CAR-TP [median: 1.3 (IQR:0.7–1.9), *p* = 0.0004] groups as compared to the EC group [median: 0.4 (IQR: 0.3–0.6)], and the IgA indices were lower in CAR-TP as compared to PAR-NC (*p* = 0.007) (Figure 1A). IgM anti-Mce1A was increased in PAR-NC [median: 1.03 (IQR:0.6–1.8)] as compared to the EC group [median: 0.5 (IQR:0.4–0.9), *p* = 0.006] and CAR-TP [median: 0.3 (IQR:0.2–0.6), *p* < 0.0001], which showed negative test results for all individuals. HHC had high antibody levels [median: 0.8 (IQR: 0.6–1.04), *p* = 0.0009] compared to treated patients (CAR-TP), proving to be a potential marker of active disease (Figure 1B). In these three groups, HHC [median: 1.2 (IQR: 0.9–1.7), *p* = 0.0003], PAR-NC [median: 1.5 (IQR: 1.1–2.0), *p* < 0.0001] and CAR-TP [median: 1.2 (IQR: 1.04–1.9), *p* < 0.0001] IgG anti-Mce1A were higher than in EC [median: 0.4 (IQR: 0.3–0.5)] (Figure 1C). The PAR-NC group had moderately higher levels of IgM anti-PGL-I [median: 0.7 (IQR: 0.3–2.5)], compared to EC [median: 0.2 (IQR: 0.1–0.5), *p* = 0.0007], HHC [median: 0.4 (IQR: 0.1–0.8), *p* = 0.03] and CAR-TP [median: 0.3 (IQR: 0.2–0.5), *p* = 0.009] (Figure 1D).

### Enzyme-Linked Immunosorbent Assay Performance of Anti-Mce1A Antibody Levels for Hansen’s Disease Diagnosis

**FIGURE 2:**
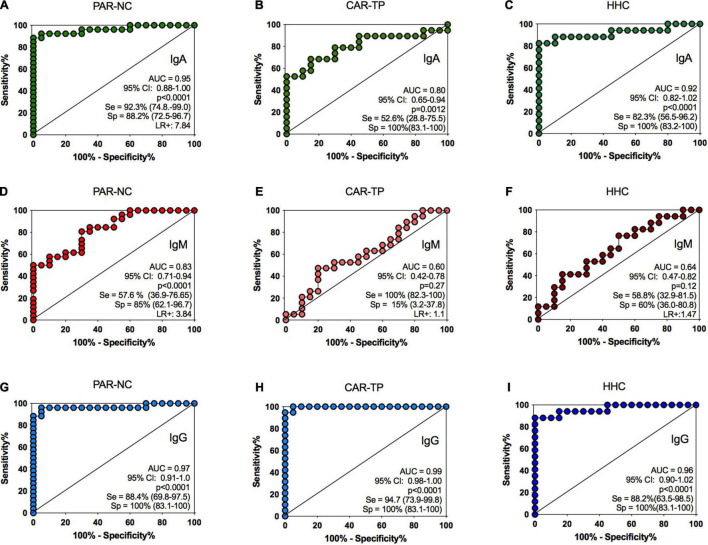
ELISA performance of anti-Mce1A antibody levels. Receiver operating characteristic analysis for comparison of IgA anti-Mce1A **(A–C)**, IgM anti-Mce1A **(D–F)**, and IgG anti-Mce1A **(G–I)** between HD patients and HHC vs. endemic controls. PAR-NC new cases of HD in Parnaíba (*n* = 26); CAR-TP patients treated in Colony Carpina (*n* = 19); HHC household contacts of HD patients in Parnaíba (*n* = 17). AUC, area under the curve; CI, confidence interval; Se, sensitivity; Sp, specificity; LR+, positive likelihood ratio.

A panel comprising plasma samples from PAR-NC, CAR-TP and HHC was examined (Table 2). The detection of IgA was strongly correlated with the PAR-NC and HHC groups (*p* < 0.0001), as well as with CAR-TP, although it was weaker (*p* = 0.0012). The IgA performance showed an area under the curve (AUC) > 0.8, sensitivity and specificity between 52.6 and 100% for treated patients and 93.2 and 88.2% for untreated patients, respectively (Figures 2A–C). In the HHC group, the test was 82.3 and 100% sensitive and specific, respectively. Additionally, the pairwise comparison of ROC curves did not show a significant difference between the IgM and APGL-I values for CAR-TP and HHC but APGL-I performance showed sensitivity and specificity of 38.4 and 100%, respectively, for untreated HD patients (*p* = 0.0002, respectively) (Table 2). The ROC curve for the IgM anti-Mce1A test did not show a significant performance, with AUCs ranging from 0.6 to 0.64 for these two groups (Figures 2E,F). The best performance of the ROC curve for IgM anti-Mce1A (AUC = 0.83) was found in the group of patients at baseline, at the time of diagnosis (PAR-NC), showing 57.6 and 85% sensitivity and specificity (*p* < 0.0001), respectively (Figure 2D). ROC curve analysis revealed excellent performance for IgG in all three groups vs. EC (*p* < 0.0001), with an AUC ≥ 0.95, sensitivity ranging from 88.2 to 94.2% and 100% specificity for all groups (Figures 2G–I).

**TABLE 2:** Comparison of receiver operating characteristic curve analysis for IgA, IgM, and total IgG against Mce1A protein and IgM anti-PGL-I in discriminating among new HD patients, treated HD patients and HHC vs. endemic controls.

Group	Antibody	AUC (95%CI)	*p*-value	Cut-off (O.D)	Sensitivity% (95%CI)	Specificity% (95%CI)	LR +
	IgA	0.95	<0.0001	0.203	93.2 (74.8–99.0)	88.2 (72.5–96.7)	7.8
	IgM	0.83	<0.0001	0.184	57.6 (36.9–76.6)	85.0 (62.1–96.7)	3.8
PAR-NC	IgG	0.97	<0.0001	0.302	88.4 (69.8–97.5)	100 (83.1–100)	−
	APGL-I	0.81	0.0002	0.295	38.4 (20.2–59.4)	100 (83.1–100)	−
	IgA	0.80	0.0012	0.203	52.6 (28.8–75.5)	100 (83.1–100)	−
	IgM	0.60	0.27	0.184	100 (82.3–100)	15 (3.2–37.8)	1.1
CAR-TP	IgG	0.99	<0.0001	0.302	94⋅7 (73.9–99.8)	100 (83.1–100)	−
	APGL-I	0.60	0.26	0.295	10.5 (1.3–33.1)	100 (83.1–100)	−
	IgA	0.92	<0.0001	0.203	82.3 (56.5–96.2)	100 (83.2–100)	−
	IgM	0.64	0.12	0.184	58.8 (32.9–81.5)	60 (36.0–80.8)	1.4
HHC	IgG	0.96	<0.0001	0.302	88.2 (63.5–98.5)	100 (83.1–100)	−
	APGL-I	0.67	0.06	0.295	11.7 (1.4–36.4)	100 (83.1–100)	−

*AUC, area under the receiver operating characteristic curve; CI, confidence interval; O.D, optical density; LR+, positive likelihood ratio; PAR-NC, new cases of HD in Parnaíba; CAR-TP, patients treated in Colony Carpina; HHC household contacts of HD patients in Parnaíba.*

### Seropositivity Pattern for New Serological Biomarkers in Hansen’s Disease

The IgA titer was positive in 92.3% of PAR-NC patients, and 84% were positive for IgG, regardless of the clinical and operational classification of the evaluated cases. The Anti-Mce1A IgM titer was positive in 50% of HD patients, while the positive APGL-I titer was detected only in 38.5%. In contrast, treated patients had no detection of anti-Mce1A IgM antibodies, and only 11.1% were positive for APGL-I. IgA and IgG titers remained positive in 52.6 and 89.5% of cases, respectively. Among the HHC, IgA and IgG antibody titers were positive in 88.2 and 64.7% of patients, respectively. The IgM titer among HHC was positive in 29.4% [index median: 0.8 (IQR: 0.6–1.04)], 17.6% better than APGL-I [index median: 0.4 (IQR: 0.1–0.8)] (Table 3).

**TABLE 3:** Positivity to antibodies against Mce1A protein and PGL-I in different groups of studies.

Groups	No of cases	IgA *n* (%)	*x^2^; p*-value	IgM *n* (%)	*x^2^; p*-value	IgG *n* (%)	*x^2^; p*-value	APGL-I *n* (%)	*x^2^; p*-value
PAR-NC	26	24 (92.3)	34.7;<0.0001	13 (50)	6.1; 0.01	22 (84.6)	32.4;<0.0001	10 (38.5)	9.8; 0.001
CAR-TP	19	10 (52.6)	10.9; 0.0009	0 (0)	3.0; 0.07	17 (89.5)	31.7;<0.0001	2 (11.1)	2.2; 0.1
HHC	17	15 (88.2)	25.9;<0.0001	5 (29.4)	1.1; 0.2	11 (64.7)	18.4<0.0001	2 (11.8)	2.4; 0.1
EC	20	1 (5.0)		3 (15)		0 (0)		0 (0)	

*Chi-squared test between HD patients and HHC vs. EC.*

*PAR-NC, new cases of HD patients Parnaíba; CAR-TP, patients treated in Colony Carpina; HHC, household contacts of HD patients in Parnaíba; EC, endemic controls.*

**FIGURE 3:**
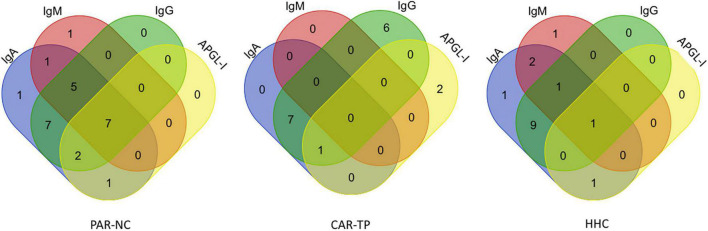
Seropositivity pattern of antibodies in HD. The Venn diagrams represent the overlap in the number of positive IgA, IgM, IgG anti-Mce1A, and APGL-I antibodies in each of the comparison groups new cases of HD in Parnaíba (PAR-NC), treated HD patients in Colony Carpina (CAR-TP) and household contacts of HD patients in Parnaíba (HHC).

The antibody titers differed for the three study groups (PAR-NC, CAR-TP, and HHC), considering that newly diagnosed patients (PAR-NC) had a greater number of positive individuals for all biomarkers tested (IgA, IgM, IgG, and APGL-I) with a predominance of positive anti-Mce1A IgM titers. Treated patients (CAR-TP) had positive titers only for IgA and IgG anti-Mce1A antibodies. APGL-I antibodies did not achieve a satisfactory response for detecting HD cases and infected HHC (Figure 3).

### Correlation Between Immunoglobulins and Mce1A Protein

**FIGURE 4:**
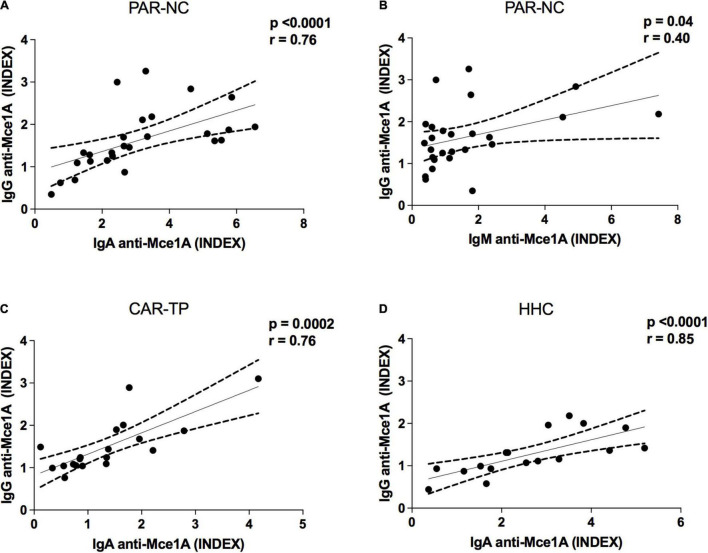
Immunoglobulins correlation profile in HD. Spearman’s correlation coefficient was used to compare immunoglobulin indices. PAR-NC new cases of HD in Parnaíba **(A,B)**; CAR-TP patients treated in Colony Carpina **(C)**; HHC household contacts of HD patients in Parnaíba **(D)**.

Correlation analyses were performed to assess the different levels of the serological markers tested. There was a strong positive correlation between the indices of anti-Mce1A IgA and IgG in the PAR-HD group (*r* = 0.76; *p* < 0.0001) (Figure 4A), CAR-TP (*r* = 0.76; *p* = 0.0002) (Figure 4C), and HHC (*r* = 0.85; *p* < 0.0001) (Figure 4D). Similarly, a moderate correlation was found between plasma anti-Mce1A IgG and IgM (*r* = 0.40; *p* = 0.04) in the new HD cases (Figure 4B).

### Logistic Regression Analysis to Evaluate the Potential of Anti-Mce1A Antibodies as Predictors of the Diagnosis of Hansen’s Disease

**FIGURE 5:**
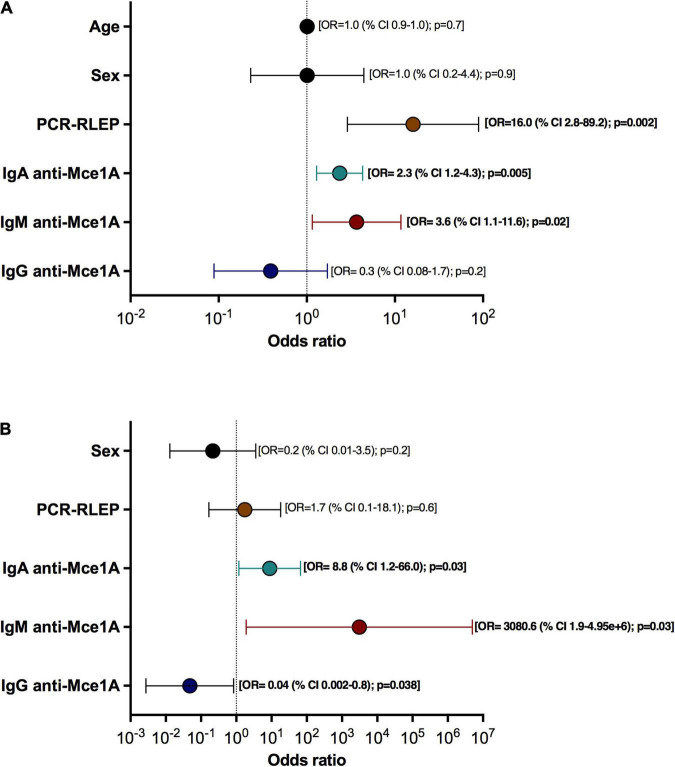
Potential of anti-Mce1A antibodies as predictors of the diagnosis of HD. Binomial logistic regression analyses showing odds ratios (ORs) and 95% confidence intervals (CIs) for HD diagnosis with independent variables: age, sex, PCR-RLEP, and antibodies against Mce1A (IgA, IgM, and IgG) for all groups (PAR-NC, CAR-TP, HHC, and EC) **(A)** and between untreated patients (PAR-NC) and treated patients (CAR-TP) **(B)**.

The logistic regression model demonstrated an association of IgM, IgA and PCR-RLEP with the clinical outcomes. (*X*^2^ = 45.8; *p* < 0.001; *R*^2^ MacFadden = 0.49; Accuracy = 0.88; Specificity = 0.927; Sensitivity = 0.80; AUC = 0.915), Patients with positive anti-Mce1A IgM titers had a 3.6 chance [OR = 3.6 (95% CI = 1.1–11.6); *p* = 0.028] and anti-Mce1A IgA titer had a 2.3 chance [OR = 2.3 (95% CI = 1.2–4.3); *p* = 0.005] of being diagnosed with HD compared to healthy volunteers. PCR-RLEP had a 16.0 chance of identifying HD [OR = 16.0 (95% CI = 2.8–89.2); *p* = 0.002]. The age [OR = 1.0 (95% CI = 0.9–1.0); *p* = 0.7) and sex [OR = 1.0 (95% CI = 0.2–4.4); *p* = 0.9] of the population did not affect the model. The positive anti-Mce1A IgG titer [OR = 0.3 (95% CI = 0.08–1.7); *p* = 0.2) was not associated with the outcome of the diagnosis for HD because this test was positive in the different groups of patients and contacts (Figure 5A). The second logistic regression model also showed the association of serological markers with the active disease (*X*^2^ = 40.5; *p* < 0.001; *R*^2^ MacFadden = 0.68; Accuracy = 0.90; Specificity = 0.94; Sensitivity = 0.88; AUC = 0.96) using treated patients (CAR-TP) and new cases (PAR-NC). The results are maintained in relation to the IgM [OR = 3.080 (95% CI = 1.9–4.95e + 6); *p* = 0.03;] and IgA [OR = 8.8 (95% CI = 1.2–66.0); *p* = 0.03] titers and clinical outcome with an increased association between these tests and the outcome of having HD. PCR-RLEP was not associated with the outcome in this model [OR = 1.7 (95% CI = 0.1–18.1); *p* = 0.6]. This result is due to the presence of positive PCR tests in the group of treated patients with high bacillary load at the time of baseline diagnosis and untreated new cases. The anti-Mce1A IgG titer was not associated with disease activity [OR = 0.04 (95% CI = 0.002–0.8); *p* = 0.038] because the tests were positive in new cases and treated patients (PAR-NC and CAR-TP) (Figure 5B). The APGL-I serology was removed from the analysis because it interfered with the performance of the logistic regression model, given the high number of negative results in common in the patient and contact groups evaluated. Age was not included in the second model due to the interference of the elderly population from the colony.

## Discussion

HD patients were compulsorily institutionalized in HD colonies before the establishment of specific treatment. Due to late diagnosis, insufficient treatment, or patients initially highly infected, colony cases of HD had a high bacillary load at diagnosis, who remained test positive by PCR in 57.9% of treated patients. Large numbers of dead *M. leprae* may persist for several years after the killing of all bacilli by effective MDT (20). The absence of diagnostic tests, especially subclinical infection, frequently leads to a delayed diagnosis, resulting in large numbers of undetected cases and not reaching the WHO target for the elimination of HD as a public health problem (21). Our anti-Mce1A serology results show they may serve as biomarkers capable of detecting cases of HD and HHCs who have not yet developed dermatoneurological classical signs and symptoms, and for monitoring treated patients.

The levels of IgA and IgG antibody titers against the Mce1A protein in the three groups we evaluated (contacts, new cases and treated patients) were significantly elevated but not among the healthy controls in endemic communities. IgM appeared to be a sensitive biomarker for identifying active disease, since no colony population treated with MDT had a positive titer. Positive IgM titer indicates a need for a robust clinical investigation of HHCs and individuals in endemic regions for HD.

The serological assays using PGL-I or LID antigens (NDO-BSA, NDO-LID, LID-1, and others) already reported present results in the literature with high seropositivity almost only in MB patients. However, anti-Mce1A antibodies demonstrate satisfactory seropositivity for the antibodies tested in both PB and MB patients and even in household contacts, as a complementary diagnostic tool capable of detecting potential cases early, as previously described by Lima et al. (15). IgG antibodies are characterized by prolonged exposure to the bacillus (22), corroborating our results of anti-Mce1A IgG ELISA with high seropositivity in new cases and treated patients with MDT. Early detection of HD cases is an important goal for disease control and elimination. Thus, the screening of different classes of immunoglobulins increases the possibility of diagnosis, with anti-Mce1A IgA being a potential biomarker in the screening of contact with *M. leprae*. In line with the findings of our study, Silva et al. (23) reports that IgA antibodies play a role in protecting against mycobacteria in the nasal mucosa and a biomarker of contact with the bacillus (23). As also, the search for an ideal serology is also associated with a satisfactory marker of disease activity. Thus, our results suggest that anti-Mce1A IgM ELISA is the indicator marker of active disease, due to the absence of positivity in treated patients. Another alternative for the use of serological tests is community screening, being positive results indicative of potential community contact with the bacillus. The presence of positive serology among endemic controls (healthy individuals without dermatoneurological signs of HD) may be associated with increased exposure in hyperendemic regions, identified in our study in 5% of the IgA ELISA and 15% of the IgM ELISA. However, further studies need to be performed to clinically follow-up these individuals. Therefore, this means that all isotypes should always be measured. We believe that those individuals that are positive for IgM or two isotypes with high indices should be clinically followed every year.

The positive anti-Mce1A IgG antibody titers (89.5%) with high diagnostic accuracy (94.7% sensitivity and 100% specificity) and negative anti-Mce1A IgM antibody titers (0%) among treated HD patients in the colony indicate the potential of these serological markers to monitor treatment response.

This group’s previous work in a population from another endemic region in the state of Bahia showed elevated IgA, IgM, and IgG titers in cases of paucibacillary and multibacillary HD. BCG vaccination and latent tuberculosis infection did not induce cross-reactive anti-Mce1A antibodies in HD patients (15). Despite the presence of Mce1A protein in the cell wall of *Mycobacterium bovis* BCG (24), no statistical difference was observed in the anti-Mce1A ELISA response between vaccinated and non-vaccinated patients (11, 25) and prior BCG vaccination does not influence antibody levels against *M. tuberculosis* proteins (26). A linear immunodominant epitope KRRITPKD (residues 131 and 138 in Mce1A) is highly conserved in *M. tuberculosis*, which is a possible explanation for the difference in response between patients with tuberculosis and Hansen’s disease, despite the homology between the mce1 gene (25). Although mce genes have been reported in many bacterial species, these genes exist as operons in mycobacteria only, hence regarded as important virulence attributes (24, 27).

The antibody response to PGL-I is the most widely evaluated biomarker for HD, and it has been shown extensively that the detection of α-PGL-I antibodies only is not sufficient to identify all HD patients, and PB cases generally lack an antibody response against PGL-I (21, 22). ELISAs targeting the PGL-I antigen showed lower sensitivity than the other antigens, but it did not affect the specificity, and a meta-analysis study showed a mean sensitivity of 59.1 (95% IC 50.6–67.1) and 91.7% (95% IC 83.9–94.9) specificity. Of all available serological tests in 78 studies, ELISA was predominantly studied, and its sensitivity varied widely from 0 to 100% and the specificity varied from 13 to 100% (28). The summary ROC plot using other antigens showed the sensitivity of PGL-I ELISA was 63.8% (95% CI 55.0–71.8), and the specificity was 91.0% (95% CI 86.9–93.9) (28). The sensitivity of quantitative polymerase chain reaction (qPCR) varied from 51 to 91%, and the specificity varied from 46 to 100%. The summary sensitivity of the RLEP test was not different from that of the other PCR targets, and the specificity was greater in studies that used RLEP as a conventional PCR target (28). These false-negative patients will not be treated, and if these patients are MB patients, then transmission could continue due to using tests with low diagnostic accuracy (29).

Summarizing and aggregating the results from our three previous published studies searching for HD involving prison male/female populations and from the community (*n* = 2,133 evaluated individuals) in São Paulo state, the authors found 112 new cases of HD, with new case detection rate (NCDR) 6.5%; macular mild lesion in 93.7%; nerve impairment on palpation in 91.9 and 67% defined as having grade 2 disability (67%). On the other hand, APGL-I titer was positive in only 31.3% of the general population, 30.3% of the non-HD group and 54.4% of HD patients, although it was officially considered high for the non-endemic state, highlighting the hidden presence and the diagnostic challenge of HD and the low sensitivity as diagnostic test for new cases and screening contacts (8, 30, 31).

Several tests have been developed to assess anti-PGL-I antibody, a known biomarker of *M*. *leprae* infection, including ELISA and lateral flow rapid tests that incorporate synthetic PGL-I (ND-O-BSA) or protein glycoconjugates, such as NDO-LID (27). However, anti-Mce1A serologic assay remedies the main gaps of the previous serologic test (PGL-I), as it demonstrates higher sensitivity, regardless of the clinical form or bacillary load, and increased seropositivity in paucibacillary cases of difficult clinical diagnosis.

Therefore, compared to the traditional clinical and other laboratory tests for HD, the anti-Mce1A serology results proved to be superior for the diagnosis of new cases of HD (including PB cases), monitoring treatment response, and identifying infected HHC of index cases.

Brazilian epidemiological indicators and the current global HD situation confirm the scenario of continued transmission and its maintenance as a public health problem that has not yet been resolved. The reality of the disease in Brazil reinforces the importance of developing new tools for the diagnosis and monitoring of HD, and studies that address the humoral immunological profile of HD patients and their contacts in addition to anti-PGL-I are rare, with results limited to multibacillary only and which is still being implemented in public health services in the country. Therefore, the development of HD diagnostic techniques in all clinical forms, both multi and paucibacillary and/or as a way of monitoring cases, in addition to their contacts and the expected search for an early diagnosis in its subclinical phase become goals to be sought to achieve the HD control goals recommended by the WHO and intended by the Ministry of Health.

In summary, in addition to understanding of the role of Mce1A in the pathogenesis of HD, it offers a highly useful target for immunological biomarker response for the implementation strategies of low-cost and easy-to-perform serological diagnostic platforms for HD. Such platforms will constitute an important technological advance for public health control of HD that can interrupt the chain of transmission of the disease, in addition to preventing deformities, disability and stigma associated with this ancient disease.

## Data Availability Statement

The original contributions presented in the study are included in the article/supplementary material, further inquiries can be directed to the corresponding author.

## Ethics Statement

The studies involving human participants were reviewed and approved by the Institutional Review Board for Human Research of the HCFMRP-USP (MH-Brazil Project—Protocol number 16620/2014). Written informed consent to participate in this study was provided by the participants’ legal guardian/next of kin.

## Author Contributions

FL and MF substantially contributed to manuscript conception and design, acquisition of data, and analysis and interpretation of data. MF, JS, RA, and EA contributed to the clinical care of patients and collected the samples. FL, VA, VB, and NP contributed to the development of experiments. FL and DT contributed to the statistical analysis and interpretation of the data. LR and SA conducted scientific guidance and advice. MF gave final approval of the final submitted version. All authors contributed to the article and approved the submitted version.

## Conflict of Interest

The authors declare that the research was conducted in the absence of any commercial or financial relationships that could be construed as a potential conflict of interest.

## Publisher’s Note

All claims expressed in this article are solely those of the authors and do not necessarily represent those of their affiliated organizations, or those of the publisher, the editors and the reviewers. Any product that may be evaluated in this article, or claim that may be made by its manufacturer, is not guaranteed or endorsed by the publisher.

## Footnotes

1
http://bioinformatics.psb.ugent.be/webtools/Venn/
